# Outcomes of total hip arthroplasty in patients with primary immune thrombocytopenia

**DOI:** 10.1186/s12891-015-0742-8

**Published:** 2015-10-05

**Authors:** Seung-Jae Lim, Ingwon Yeo, Chan-Woo Park, Young-Wan Moon, Youn-Soo Park

**Affiliations:** Department of Orthopedic Surgery, Samsung Medical Center, Sungkyunkwan University School of Medicine, 81 Irwon-ro, Gangnam-gu, Seoul, 135-710 South Korea

**Keywords:** Total hip arthroplasty, Immune thrombocytopenia, Transfusion, Outcomes

## Abstract

**Background:**

Immune thrombocytopenia (ITP) is an immune-mediated acquired disease that is characterized by a decrease in the platelet count and an increased risk of bleeding. There is little information in the literature about the results of major joint replacement surgery in patients with ITP. The aim of this study was to report on the results of total hip arthroplasty (THA) in patients with primary ITP.

**Methods:**

We retrospectively identified 15 THAs performed in 11 patients with primary ITP. The study group was matched (1:2) to a non-ITP control group of 30 THAs in 22 patients. According to the perioperative hematologic evaluation, blood management interventions were performed. All procedures were performed by a single surgeon and all patients received cementless components with ceramic-on-ceramic bearing. Mean duration of follow-up was 7.1 years (range, 2–13).

**Results:**

No significant differences were found between the two groups with regard to mean operative time, intraoperative blood loss, amount of closed suction drainage, length of hospital stay, and readmission rate. However, the proportion of patients requiring transfusion of packed red blood cells and/or platelet concentrate was higher in the ITP group when compared to the non-ITP group. Mean Harris hip score improved from 49.5 points preoperatively to 93.4 points at the final follow-up and no hips were revised for loosening or osteolysis in the ITP group. No significant differences were found between the two groups with respect to mean postoperative Harris hip scores and complication rates.

**Conclusions:**

Our study showed encouraging clinical and radiographic results of THA in patients with ITP without increased risk of adverse events compared to those in patients without ITP. On the basis of these findings, we suggest that modern cementless THA might be a viable treatment for achieving functional improvement in patients with ITP and end-stage hip disease.

## Background

Immune thrombocytopenia (ITP) is an immune-mediated acquired disease characterized by a transient or persistent decrease in platelet count and an increased risk of bleeding [1–3]. According to the International Working Group (IWG) criteria, primary ITP is defined as an autoimmune disorder characterized by isolated thrombocytopenia (peripheral blood platelet count of <100 × 10^9^/L) in the absence of other causes or disorders that might be associated with thrombocytopenia [1]. Corticosteroids are widely accepted as the most appropriate first-line treatment for ITP patients [2, 3], and in such patients, the prevalence of steroid-induced avascular necrosis lies between 9 % and 40 % with the femoral head being the most commonly affected site [4]. Furthermore, the prevalence of osteoarthritis of the hip is increasing [5, 6], and thus, the number of elderly ITP patients requiring total hip arthroplasty (THA) can be expected to rise.

Patients with ITP undergoing surgery are at increased risk of adverse perioperative events, particularly if blood or blood product transfusions are required preoperatively, or if the surgical procedure is performed on an emergency basis [7]. Furthermore, because greater perioperative blood loss may be expected in patients with ITP undergoing THA, and because of the additional risk of periprosthetic infection, special medical management is required to minimize complications in patients with ITP undergoing THA. However, little information is available in the literature concerning the results of major joint replacement surgery in patients with ITP, being limited to isolated English and non-English language case reports [8–10]. Accordingly, questions remain as to whether patients with ITP can safely undergo THA, as these patients may be at increased risk of surgical complications and mortality [7, 11].

Therefore, the primary aim of this study was to report on the clinical and radiographic outcomes of THA in patients with primary ITP, and the secondary aim was to determine whether patients with and without ITP differ with respect to early postoperative outcomes, Harris hip scores, and radiographic results.

## Methods

We retrospectively reviewed the medical records of 11 consecutive patients with primary ITP who underwent 15 cementless THAs between 2001 and 2011 at our institution. Primary ITP was diagnosed according to the IWG criteria [1] after thorough hematologic evaluation, including consultation with a hematologist. All THA candidates underwent complete blood count screening. In patients with a low platelet count (<100 × 10^9^/L) the following investigations were performed: reticulocyte counts, estimation of red blood cell and thrombocyte indices, bone marrow cytology (if thrombocytopenia had persisted for > 6 months), thyroid stimulating hormone (TSH), aspartate aminotransferase (AST), alanine aminotransferase (ALT), gamma-glutamyl transpeptidase (GGT), creatinine, blood urea nitrogen (BUN), lactate dehydrogenase (LDH), bilirubin, Coombs test, antiphospholipid antibodies, immunoglobulin subclass analysis, von Willebrand antigen and ristocetin cofactor, and serologic tests for hepatitis B and C, human immunodeficiency virus (HIV), Epstein-Barr virus (EBV), and cytomegalovirus (CMV). Two of the eleven patients were diagnosed with ITP as a result of the preoperative hematologic evaluation. The other nine had already been diagnosed with chronic ITP and had been previously managed by a hematologist. None of the patients with ITP were lost to follow-up, and all were included in this study. This study protocol was approved by the Review Board of Samsung Medical Center. Written informed consent for participation in the study was obtained from all patients.

Of the 11 patients (15 hips), 2 (2 hips) were male and 9 (13 hips) were female and they had an overall mean age of 49.3 years (range, 35–68 years) at the time of THA. The mean time elapsed between ITP diagnosis and surgery was 4.3 years (range, 0–13 years), and mean body mass index (BMI) was 24.2 kg/m^2^ (range, 17.2–28.6 kg/m^2^). Twelve hips (8 patients) were replaced because of osteonecrosis of the femoral head, and 3 hips (3 patients) were replaced because of osteoarthritis. The mean American Society of Anesthesiologists (ASA) score was 1.7 (range, 1–3), and mean follow-up was 7.1 years (range, 2–13 years).

The 11 patients with ITP (15 hips) were matched (1:2) with 22 patients without ITP (30 hips) with respect to: patient age (±1 year), sex, preoperative diagnosis (osteonecrosis or osteoarthritis), and operating surgeon. Demographic data of the ITP and non-ITP groups are summarized in Table 1. No significant differences were evident between the two groups with respect to age, sex, BMI, etiology of THA, ASA scores, type of anesthesia, or follow-up period (*p* > 0.05 for all).

**Table 1:** Demographics of the ITP and non-ITP groups

	ITP Group	Non-ITP Group	*p* value
Number of patients (hips)	11 (15)	22 (30)	
Mean age (years)	49.3 (35–68)	49.0 (35–68)	0.317
Sex (male/female)	2/13	4/26	1.000
Mean body mass index (kg/m^2^)	24.2 (17.2–28.6)	24.7 (18.9–31.3)	0.460
Etiology			1.000
Osteonecrosis	12	24	
Osteoarthritis	3	6	
Mean ASA score	1.7 (1–3)	1.4 (1–3)	0.160
Type of anesthesia			0.331
General	7	9	
Spinal	8	21	
Mean duration of follow-up (years)	7.1 (2–13)	7.2 (2–13)	0.285

*ITP* immune thrombocytopenia, *ASA* American Society of Anesthesiologists

In the ITP group, preoperative mean serum hemoglobin was 11.7 g/dL (range, 7.7–15.1 g/dL) and mean platelet count was 92.7 × 10^9^/L (range, 34 × 10^9^/L-149 × 10^9^/L). At time of surgery, mean platelet count was more than 100 × 10^9^/L in 4 patients (5 hips) and less than this in 7 patients (10 hips). Packed red blood cells (pRBCs), platelet concentrate (PC), and intravenous immunoglobulin (IVIG) were transfused preoperatively based on the results of hematological studies and hematologic consultations (Table 2). During the study period, we used the same transfusion thresholds for both pRBCs and PC and/or IVIG. A hemoglobin level of 8 g/dL was the transfusion threshold for pRBCs [12, 13]. A platelet count of less than 80 × 10^9^/L was the threshold for PC transfusion and/or IVIG administration [14]. In the non-ITP control group, mean preoperative serum hemoglobin was 12.6 g/dL (range, 10.9-15.6 g/dL) and mean platelet count was 286 × 10^9^/L (range, 111 × 10^9^/L-357 × 10^9^/L). No patient in the non-ITP group required a blood transfusion preoperatively. No significant difference was found between the two groups in preoperative serum hemoglobin level (*p* = 0.211), but preoperative platelet count was significantly lower in the ITP group (*p* = 0.001) than that in the non-ITP group.

**Table 2:** Perioperative Transfusions in the ITP Group

Case	Diagnosis	ASA	PLT (×10^9^/)	Hb (g/dL)	Preoperative transfusion (units)	Postoperative transfusion (units)
1	ON	1	83	10.5	-	PC, 4, pRBC 2
2	ON	2	98	11.3	-	PC 6, pRBC 2
3	ON	3	52	7.7	PC 6, pRBC 2	PC 6, pRBC 4
4	OA	1	72	13.4	PC 8	-
5	OA	2	65	10.9	IVIG	PC 8, pRBC 2
6	OA	2	71	13.4	PC 15	
7	ON	3	55	11.7	IVIG, PC 12	
8	ON	2	99	13.1	-	pRBC 2
9	ON	2	131	14.1	-	PC 8
10	ON	1	131	15.1	-	-
11	ON	2	61	12.7	IVIG, PC 8	pRBC 2
12	ON	1	34	11.8	IVIG, PC 12	PC 8, pRBC 3
13	ON	2	143	9.4	-	
14	ON	1	149	10.0	-	pRBC 2
15	ON	1	146	10.8	-	-
Mean		1.7	92.7	11.7	-	-

*ASA* American Society of Anesthesiologists, *PLT* platelet, *Hb* haemoglobin, *ON* osteonecrosis, *OA* osteoarthritis, *PC* platelet concentrate, *pRBC* packed red blood cell, *IVIG* intravenous immunoglobulin

### Operative treatment

**Fig. 1:**
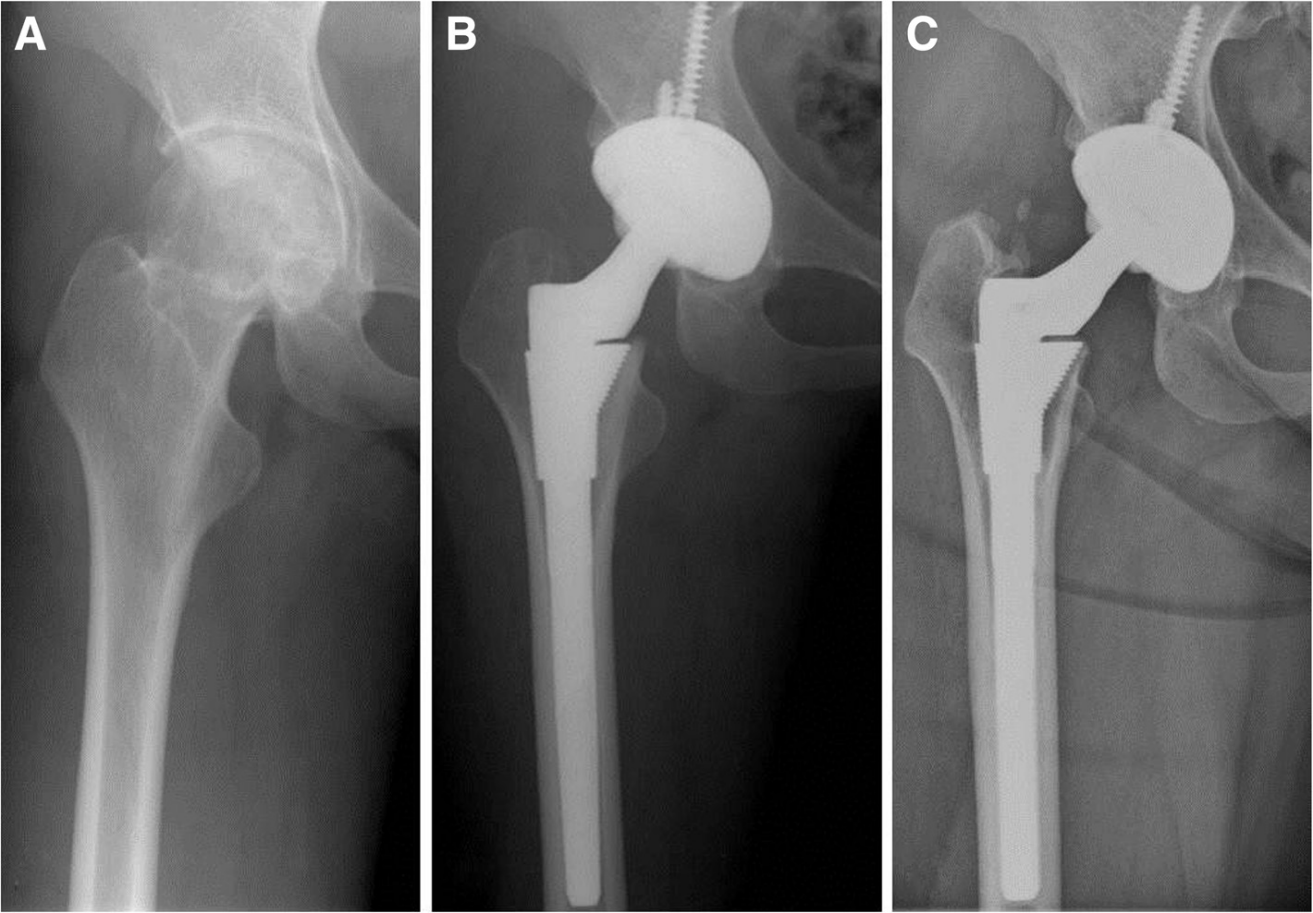
**a** Pre-operative radiograph showed osteonecrosis of the femoral head in patient with primary immune thrombocytopenia. **b** Preoperative transfusions of six units of platelet concentrate and two units of packed red blood cells were performed. Postoperatively, six units of platelet concentrates and four units of packed red blood cells were transfused. Post-operative radiograph of cementless total hip arthroplasty performed with ceramic-on-ceramic bearing. **c** Twelve-year follow-up radiograph showed a well-fixed prosthesis with no osteolysis

All the operations were performed by a single surgeon in the lateral position through an anterolateral approach. Cementless components were used in all hips in both groups. In the ITP group, the acetabular components used were as follows; Bencox® cups (Corentec, Seoul, Korea; *n* = 7), Duraloc® Option cups (DePuy/J&J, Warsaw, IN, USA; *n* = 6), and SPH® Contact cups (Lima, Udin, Italy; *n* = 2). The femoral components used were Bencox® stems (Corentec, Seoul, Korea; *n* = 7), S-ROM® stems (DePuy/J&J, Warsaw, IN, USA; *n* = 6), and C2® stems (Lima, Udin, Italy; *n* = 2). In all 22 hips, an alumina ceramic head and liner (Biolox® Forte, CeramTec AG, Plochingen, Germany; *n* = 11 and Biolox® Delta, CeramTec AG, Plochingen, Germany; *n* = 4) were used (Fig. 1). Twenty-eight millimeter heads were used in 10 hips, 32 mm in 4 hips, and 36 mm in 1 hip. In the non-ITP group, the acetabular components used were as follows; Bencox® cups (Corentec, Seoul, Korea; *n* = 9), Duraloc® Option cups (DePuy/J&J, Warsaw, IN, USA; *n* = 17), and SPH® Contact cups (Lima, Udin, Italy; *n* = 4). The femoral components used were Bencox® stems (Corentec, Seoul, Korea; *n* = 9), S-ROM® stems (DePuy/J&J, Warsaw, IN, USA; *n* = 17), and C2® stems (Lima, Udin, Italy; *n* =4). In all 30 hips, an alumina ceramic head and liner (Biolox® Forte, CeramTec AG, Plochingen, Germany; *n* = 21 and Biolox® Delta, CeramTec AG, Plochingen, Germany; *n* = 9) were used. Twenty-eight millimeter heads were used in 18 hips, 32 mm in 11 hips, and 36 mm in 1 hip. Intra-operative blood loss was estimated by measuring the volume of blood in the suction bottle, by weighing mops and gauze pieces used during surgery, and by visual estimation of loss in the operative field. Hemostatic agents such as tranexamic acid or fibrin sealant were not used during the study period. Closed suction drains were routinely used in all patients.

### Postoperative treatment

Both groups received the same postoperative management, except that routine hematological tests were performed more frequently in the ITP group. Drains were removed 48 h after the operation and the drainage volume was recorded as the post-operative blood loss. Mechanical compression devices alone were used for deep vein thrombosis (DVT) prophylaxis in both groups without additional pharmacological thromboprophylaxis because the prevalence of venous thromboembolism in Korean patients without chemoprophylaxis had been reported to be low at that time [15]. Antibiotic prophylaxis was administered to all patients starting 1 hour before surgery, continuing until 3 days after surgery. Postoperatively, hematological studies were performed in all patients until hemoglobin and platelet levels reached and remained at or above preoperative levels. Transfusions were performed based on the results of hematological studies and amounts of blood collected via suction drains. The transfusion thresholds were hemoglobin levels of 8 g/dL for pRBCs, and platelet count of 80 × 10^9^/L for PC for the entire postoperative period. All patients received the same postoperative rehabilitation. All were allowed to stand on the second or third post-operative day and to progress to partial weight bearing with crutches as tolerated. Patients were allowed full weight-bearing after 4 to 6 weeks.

### Assessments

A single independent observer, who had not participated in the operations, retrieved the following clinical data by reviewing archived records: operative times, intraoperative estimated blood losses, closed suction drainage amounts, postoperative blood product transfusions (pRBC and PC), durations of hospital stay, readmission rates, and complications. All patients were examined clinically at latest follow-up visits. Harris hip scores [16] were used to assess pain levels and function. Radiographic assessments were performed immediately after surgery, and then at 6 weeks, 3 and 6 months, and at 1 year postoperatively, and then at 1 year or 2 year intervals until final follow-up visits. Radiographic evaluations were performed by the same independent observer. The 6 week postoperative radiographs were used as references for radiographic evaluations of component stability, wear, osteolysis, and loosening [17]. The stability of fixation of femoral and acetabular components were classified as previously described [18, 19].

### Statistical analysis

Statistical analyses were performed using IBM SPSS Statistics version 22.0 (IBM Corp., Somers, NY, USA). The paired t test or the Wilcoxon matched pairs test was used to analyze continuous variables, and McNemar's chi square test was used to analyze dichotomous values. Statistical significance was accepted for *p* values < 0.05.

## Results

No significant differences were found between the ITP and non-ITP groups with respect to mean operative time, intraoperative estimated blood loss, closed suction drainage amount, duration of hospital stay, or readmission rates (*p* > 0.05 for all). However, the two groups differed in terms of postoperative blood management interventions. The pRBC transfusion rate was higher in the ITP group (*p* = 0.003), and the PC transfusion rate and total number of units of PC transfused were higher in the ITP group (*p* = 0.001 for both). On the other hand, the total number of units of pRBC transfused were not significantly different between the two groups (*p* = 0.054),

In the ITP group, the mean Harris hip score improved from 49.5 points (range, 37–61 points) to 93.4 points (range, 90–100 points) at final follow-up. No significant intergroup differences in postoperative Harris hip scores were seen between groups (*p* = 0.163). In the ITP group, no femoral or acetabular component showed radiographic evidence of mechanical loosening, and no component had been revised at final follow-up. Furthermore, no osteolysis was observed, and no significant differences were found between the two groups with respect to component stability, osteolysis, or revision (*p* = 1.000 for all).

In the ITP group, no hip developed wound complications, deep infection or symptomatic venous thromboembolism during follow-up, and no death occurred. Furthermore, no ceramic fracture or squeaking was encountered. Complication rates were identical in the two groups (*p* = 1.000).

Clinical and radiographic outcome data for both groups are summarized in Table 3.

**Table 3:** Outcome data of the ITP and non-ITP groups

	ITP Group (*N* = 15)	Non-ITP Group (*N* = 30)	*p* value
Mean operative time (minutes)	68.8 (49–104)	61.3 (43–104)	0.550
Mean intraoperative estimated blood loss (mL)	401 (100–1030)	375 (200–850)	0.115
Mean amount of suction drainage (mL)	526 (150–1040)	545 (220–980)	0.394
Number of patients transfused postoperatively			
pRBC (%)	8 (53.3)	4 (13.3)	0.003
PC (%)	6 (40.0)	0 (0.0)	0.001
Mean number of units transfused postoperatively			
pRBC	1.27 (0–4)	0.35 (0–4)	0.054
PC	2.67 (0–8)	0	0.001
Mean length of hospital stay (days)	9.5 (7–14)	9.2 (7–14)	0.445
Readmission rate (%)	0	0	1.000
Mean postoperative Harris hip score (points)	93.4 (90–100)	94.9 (92–100)	0.163
Number of patients experiencing post-operative complications			
Component loosening	0	0	1.000
Osteolysis	0	0	1.000
Wound hematoma	0	0	1.000
Wound infection	0	0	1.000
Deep infection	0	0	1.000
Venous thromboembolism	0	0	1.000
Death	0	0	1.000

*ITP* immune thrombocytopenia, *pRBC* packed red blood cells, *PC* platelet concentrate

## Discussion

Several previous studies demonstrated significantly higher overall complication rates after a range of procedures among patients with ITP, who were found to have considerably higher risks of acute renal failure, postoperative bleeding, septicemia, and pneumonia [7, 11]. Thus, at the onset of this study, we postulated that major joint replacement surgery in patients with ITP might be associated with higher risks of perioperative bleeding, wound complications or periprosthetic infection. However, only limited information was available about the outcomes of major orthopedic surgery in patients with ITP [8–10]. Nezu et al. described the case of a 42-year-old with refractory ITP who underwent hip replacement under the cover of vinca alkaloids and colchicine after their platelet count was not sufficiently elevated by first or second line therapy [8]. Kim et al. described a series of five patients who underwent total hip replacement for osteonecrosis of femoral head associated with underlying ITP [9]. Singhal et al. report the case of a 61-year-old with refractory ITP who underwent total knee replacement [10]. However, no case–control studies have been reported to date concerning on patients with ITP who have undergone THA, and no specific guidelines have been issued regarding the perioperative management of such patients. Therefore, in the present study, we retrospectively analyzed the clinical and radiographic results of THA in this patient population. In particular, we analyzed operative times, intraoperative estimated blood losses, closed suction drainage amounts, postoperative blood product transfusions, hospital stay durations, re-admission rates, and complications.

Most reports of the surgical treatment of patients with ITP describe their management in the context of abdominal surgery [11], which is generally characterized by excellent intra-operative hemostasis. However, during THA, difficult to control intraoperative and postoperative bone bleeding is inevitable, and greater perioperative blood loss can be expected. Nevertheless, in the present study, perioperative blood losses, as determined by intraoperative estimated blood losses and closed suction drainage amounts, were not significantly different in the ITP and non-ITP groups. We believe that this favorable outcome might have resulted from relatively short operative times and thorough perioperative blood management interventions. All patients with ITP received pre-operative transfusions/IVIG treatment if found to be at high bleeding risk. The transfusion threshold for pRBCs was a hemoglobin level of 8 g/dL, and for PC transfusion and/or IVIG administration was a platelet count of 80 × 10^9^/L over the entire perioperative period. Furthermore, we used mechanical compression devices only alone for DVT prophylaxis, without additional pharmacological thromboprophylaxis. These blood management strategies were associated with excellent immediate postoperative clinical courses and hospital stays similar to those observed in the control group. However, an updated meta-analysis of randomized trials showed that a restrictive transfusion strategy using a hemoglobin transfusion trigger of < 7 g/dL results in a significant reduction in acute coronary syndrome, pulmonary edema, rebleeding, infections, and total mortality, compared with a more liberal strategy [20]. Thus, if more restrictive transfusion strategies had been used in the present study, some of the patients in both groups might not have needed the transfusions following THA. In addition, new cost-effective blood management tools, such as tranexamic acid or fibrin sealant, could be a promising approach to reduce bleeding and consequently lead to lower transfusion rates after total joint arthroplasty in these patient populations [21, 22].

We found that the mean Harris hip score was 93.4 points (range, 90 to 100 points) at a mean follow-up of 7.1 years (range, 2–13 years) following cementless ceramic-on-ceramic THA in patients with ITP. No abnormal radiographic changes such as loosening or osteolysis were observed at last follow-up, and no component required revision. The excellent clinical and radiographic outcomes achieved in the present study might be due to the use of ceramic-on-ceramic articulation combined with modern cementless THA prostheses, which corroborates the findings of recent clinical studies [23, 24].

Several limitations of the present study warrant consideration. First, the study is limited by its retrospective nature and by the small number of patients over a long period of enrollment. These limitations might make it difficult to accurately assess what the outcomes are of this patient population. However, these limitations were unavoidable owing to the rarity of ITP and the consequent low number of patients with this condition who undergo THA. Second, follow-up durations varied widely. While all patients were followed for a minimum of 2 years some were followed for as much as 13 years, and the average follow-up was 7.1 years. Third, the variety of implants used might have acted as a confounding factor. Nevertheless, our study is unique in that it involves a consecutive series of ITP patients treated using modern cementless components in combination with ceramic-on-ceramic bearings by a single surgeon at a single institution. Fourth, contemporary blood management strategies including the use of tranexamic acid or fibrin sealant, and more restrictive blood transfusion strategies were not used during the study period and could further influence the results.

## Conclusion

The present study describes encouraging early postoperative outcomes of THA in patients with ITP, with no increase in the risk of adverse events as compared with THA in patients without ITP. In addition, excellent clinical and radiographic results were obtained for all fifteen cementless THAs performed in eleven ITP patients. Based on these findings, we believe that modern cementless THA offers a viable means of achieving functional improvements in patients with ITP and end-stage hip disease without markedly increased perioperative risk. In addition, we hope that our findings provide a reference for surgeons who undertake major surgical interventions in in patients with ITP.

## Abbreviations

ALT: Alanine aminotransferase
ASA: American society of anesthesiologists
AST: Aspartate aminotransferase
AVN: Avascular necrosis of the femoral head
BUN: Blood urea nitrogen
CMV: Cytomegalovirus
DVT: Deep vein thrombosis
EBV: Epstein-Barr virus
GGT: Gamma-glutamyl transpeptidase
HIV: Human immunodeficiency virus
Hb: Hemoglobin
ITP: Immune thrombocytopenia
IVIG: Intravenous immunoglobulin
LDH: Llactate dehydrogenase
OA: Osteoarthritis
PC: Platelet concentrate
PLT: Platelet
pRBC: Packed red blood cell
THA: Total hip arthroplasty
TSH: Thyroid stimulating hormone
